# Ischemic and Bleeding Outcomes According to the Academic Research Consortium High Bleeding Risk Criteria in All Comers Treated by Percutaneous Coronary Interventions

**DOI:** 10.3389/fcvm.2021.620354

**Published:** 2021-12-02

**Authors:** Daphné Doomun, Ianis Doomun, Sara Schukraft, Diego Arroyo, Selma Cook, Tibor Huwyler, Peter Wenaweser, Jean-Christophe Stauffer, Jean-Jacques Goy, Mario Togni, Serban Puricel, Stéphane Cook

**Affiliations:** Department of Cardiology, University and Hospital Fribourg, Fribourg, Switzerland

**Keywords:** high bleeding risk, ACR-HBR criteria, percutaneous coronary intervention, antithrombotic therapy, major criteria, minor criteria

## Abstract

**Background:** The Academic Research Consortium have identified a set of major and minor risk factors in order to standardize the definition of a High Bleeding Risk (ACR-HBR).

**Aims:** The aim of this study is to stratify the bleeding risk in patients included in the Cardio-Fribourg registry, according to the Academic Research Consortium for High Bleeding Risk (ACR-HBR) definition, and to report ischemic and hemorrhagic events at 2-year of clinical follow-up.

**Methods:** Between 2015 and 2017, consecutive patients undergoing percutaneous coronary intervention were prospectively included in the Cardio-Fribourg registry. Patients were considered high (HBR) or low (LBR) bleeding risk depending on the ARC-HBR definition. Primary endpoints were hierarchical major bleeding events as defined by the Bleeding Academic Research Consortium (BARC) grade 3–5, and ARC patient-oriented major adverse cardiac events (POCE) at 2-year follow-up.

**Results:** Follow-up was complete in 1,080 patients. There were 354 patients in the HBR group (32.7%) and 726 patients in the low-bleeding risk (LBR) group (67.2%). At 2-year follow-up, cumulative BARC 3–5 bleedings were higher in HBR (10.5%) compared to LBR patients (1.5%, *p* < 0.01) and the impact of HBR risk factors was incremental. At 2-year follow-up, POCE were more frequent in HBR (27.4%) compared to LBR group (18.2%, <0.01). Overall mortality was higher in HBR (14.0%) vs. LBR (2.9%, *p* < 0.01).

**Conclusions:** ARC-HBR criteria appropriately identified a population at a higher risk of bleeding after percutaneous coronary intervention. An increased risk of bleeding is also associated with an increased risk of ischemic events at 2-year follow-up.

## Introduction

Dual-antiplatelet therapy (DAPT) is mandatory after percutaneous coronary intervention (PCI) but increases the risk of bleeding (1, 2). The type and duration of DAPT are balanced according to the thrombotic and bleeding risks (3). Patients at high thrombotic and low hemorrhagic risk benefit from extended DAPT, whereas patients at high bleeding but low ischemic risk do better with shorter DAPT durations (4, 5). Over the years, patient and procedural factors that increase the thrombotic risk have been identified, amongst which: diabetes mellitus, renal failure, acute coronary syndromes, heavily calcified lesions, bifurcation, extensive stent length, small stent diameter, presence of incomplete stent expansion or apposition (6–8).

Recently, the Academic Research Consortium for High Bleeding Risk (ACR-HBR) identified key factors associated with bleeding risk post PCI (9). The ARC-HBR definition is dichotomous and defines HBR as a Bleeding Academic Research Consortium (BARC) 3 or 5 bleeding risk of > 4% and/or risk of intracranial hemorrhage > 1% within 1 year after PCI. These key factors are stratified into major criteria and minor criteria (9, 10). There is emerging evidence on the discriminatory capacity and predictability of this new definition (11–13). However, the prognostic value of ARC-HBR criteria for bleeding events beyond 1 year is poorly described.

Therefore, we planned to report (a) the bleeding outcomes at 2-years of patients enrolled in the all-comers Cardio-Fribourg registry based on presence or absence of ARC-HBR major and minor risk factors, and (b) the ischemic outcomes, as HBR has to be balanced with the thrombotic risk.

## Methods

### Study Population and Data Collection

The Cardio-FR is a single-center, all-comers registry. All patients who underwent PCI at our institution between June 2015 and July 2017 and gave informed consent were included. The only exclusion criterion was the inability to sign the informed consent and/or unwillingness to participate in clinical follow-up. The indication for PCI was based on established European guidelines (14). There were no limitations on the type, number or length of the lesions treated. Treatment modalities and antithrombotic management were at the physician's discretion and according to the local standard of care at the time of intervention. Clinical follow-up was scheduled at 1, 2, 5 and 10 years. The Cardio-FR registry complied with the Helsinki Declaration and was approved by the local ethics committee (003-REP-CER-FR). All patients provided written informed consent.

### Definition of High-Bleeding Risk

Patients were considered high (HBR) or low (LBR) bleeding risk depending on the definition of ARC-HBR. The definition being dichotomous, if the patient did not meet the criteria to be classified as HBR, he fell into the LBR classification. So, in fact, the LBR group does not only correspond to patients at low risk of bleeding but also to patients at medium risk of bleeding. The ARC-HBR definition considered HBR as a risk of major (BARC 3 to 5) bleeding of ≥4% or risk of intracranial hemorrhage of ≥1% at 1 year. The major and minor criteria were described by Urban et al. (9) and are summarized in Table 1. Patients are at HBR if at least 1 major or 2 minor criteria are met. The bleeding risk was assessed at time of PCI.

**Table 1:** Distribution of HBR-ARC criteria.

	**All** **N= 1,080**	**HBR** **N= 354**	**LBR** **N= 726**	***p-*value**
**Major HBR criteria**				
Long-term OAC, n (%)	130 (12.0)	130 (36.7)	0 (0)	<0.01
eGFR <30 ml/min, n (%)	24 (2.2)	24 (6.8)	0 (0)	<0.01
Hemoglobin < 11 g/dL, n (%)	55 (5.1)	55 (15.5)	0 (0)	<0.01
Recent or recurrent major bleeding, n (%)	1 (0.1)	1 (0.3)	0 (0)	0.33
Platelet count <100 G/L, n (%)	12 (1.1)	12 (3.4)	0 (0)	<0.01
Chronic bleeding diathesis, n (%)	0 (0)	0 (0)	0 (0)	na
Liver cirrhosis with portal hypertension, n (%)	4 (0.4)	4 (1.1)	0 (0)	<0.01
Active malignancy < 12 months, n (%)	19 (1.8)	19 (5.4)	0 (0)	<0.01
Previous spontaneous ICH (any time), previous traumatic ICH < 12 months, presence of bAVM, major ICH < 6 months, n (%)	5 (0.5)	5 (1.4)	0 (0)	<0.01
Non-deferrable major surgery on DAPT, n (%)	6 (0.6)	6 (1.7)	0 (0)	<0.01
Recent major surgery or major trauma <30 days before PCI, n (%)	0 (0)	0 (0)	0 (0)	na
**Minor HBR criteria**				
Age ≥ 75 years, n (%)	282 (26.1)	211 (60.0)	71 (9.8)	<0.01
eGFR 30–59mL/min, n (%)	213 (19.7)	183 (51.7)	30 (4.1)	<0.01
Hemoglobin 11–12.9 g/dL for men and 11–11.9 g/dL for women, n (%)	150 (13.9)	108 (30.5)	42 (5.8)	<0.01
Spontaneous major bleeding not meeting the major criterion, n (%)	0 (0)	0 (0)	0 (0)	na
Long-term use of oral NSAIDs or steroids, n (%)	60 (5.6)	41 (11.6)	19 (2.6)	<0.01
Any ischemic stroke > 6 months, n (%)	45 (4.2)	31 (8.8)	14 (1.9)	<0.01

*bAVM, brain arteriovenous malformation; DAPT, dual antiplatelet therapy; eGFR, eGFR, estimated glomerular filtration rate; HBR, high bleeding risk; LBR, low bleeding risk, ICH, intracerebral hemorrhage; NSAIDs, Non-steroidal anti-inflammatory drugs; OAC, oral anticoagulation. eGFR: the closest plasma creatinine value before the procedure was taken, excluding values that may be consistent with acute renal failure; Hemoglobin: same reasoning as for eGFR; Platelet count: same reasoning as for eGFR and Hb; Non-deferrable major surgery on DAPT: we considered that any patient who had surgery that was done under DAPT was assigned this major criterion*.

### Clinical Endpoints

Clinical endpoints were reported at 2-year follow-up. Bleeding was defined as per BARC definition, with type 3 and 5 considered “major bleeding” (15). Ischemic outcomes were defined as a patient-oriented composite endpoint (POCE): all-cause mortality, any myocardial infarction, any coronary revascularization. In-hospital events were directly reported to the database, while post-discharge events were recorded by research nurses by iterative telephone calls during follow-up. These events were finally adjudicated by a clinical event committee for the present analysis.

### Statistical Analysis

Categorical variables are reported as counts and percentages; continuous variables are reported as mean and SD. Normality was assessed by visual inspection of histograms and the computation of Q-Q plots. Continuous variables are analyzed using the Student *t*-test or the Wilcoxon rank-sum test per distribution. Categorical variables were compared using chi-square or Fisher exact test as appropriate. Survival free from the occurrence of clinical endpoints was assessed by computation of Kaplan-Meier curves. Survival was compared using the log-rank test. Furthermore, clinical outcome was reported as Kaplan-Meier failure estimations. Hazard ratios are derived from univariate Cox regression.

To better illustrate the incremental risk of bleeding conditioned by an increasing number of ACR-HBR criteria per patient, we performed subgroup analysis dividing patients into groups according to the number of HBR criteria. Every subgroup was then univariately compared to the reference group consisting of patients without any ACR-HBR criteria using Cox Regression. Hazard ratios reflect the incremental risk for patients according to the number of ACR-HBR criteria compared to the reference group (i.e., patients without any criteria).

As a supplementary analysis we performed multivariate Cox Regression to identify which individual components of the HBR-Score were most strongly associated with the occurrence of bleeding in our sample. We performed backward stepwise selection separately for major and minor criteria initially saturating the model with all relevant variables. The removal criterion for the final model was *p* >0.10.

All statistical analyses were performed using dedicated software (StataCorp LP, College Station, Texas) at a 2-tailed significance level of alpha = 0.05.

## Results

### Baseline Characteristics

Of the 1,080 patients enrolled in the registry, 354 (32.7%) fulfilled the HBR definition and the remaining 726 (67.2%) were considered LBR (Supplementary Figure 1). HBR criteria and distribution are summarized in Table 1, and baseline characteristics are found in Table 2. The most frequent major HBR criterion was oral anticoagulation (OAC) (36.7%). Patients in the HBR group were older (76 [70–82] vs. 63 [56–70], *p* < 0.01), with more women (29.7% vs. 20.8%, *p* < 0.01), a higher incidence of arterial hypertension (72.0 vs. 56.3%, *p* < 0.01) but a lower incidence of positive family history for cardiovascular events (15.6 vs. 23.8%, *p* < 0.01) and less current smoker (17.8 vs. 33.3%, *p* < 0.01).

**Table 2:** Baseline characteristics.

	**All** ***N* = 1,080**	**HBR** ***N* = 354**	**LBR** ***N* = 726**	***p-*value**
Age, year [IQR]	67 [58–75]	76 [70–82]	63 [56–70]	<0.01
Male, n (%)	824 (76.3)	249 (70.3)	575 (79.2)	<0.01
Hypertension, n (%)	664 (61.5)	255 (72.0)	409 (56.3)	<0.01
Diabetes, n (%)	263 (24.4)	103 (29.1)	160 (22.0)	0.01
Insulin-dependent, n (%)	78 (7.2)	35 (9.9)	43 (5.9)	0.02
Smoking, n (%)	305 (28.2)	63 (17.8)	242 (33.3)	<0.01
Dyslipidemia, n (%)	501 (46.4)	155 (43.8)	346 (47.7)	0.24
BMI, kg/m^2^ [IQR]	27.0 [24.2–29.8]	26.2 [23.6–29.4]	27.2 [24.7–29.9]	<0.01
eGFR, mL/min [IQR]	82.2 [62.3–107.2]	56.9 [43.1–77.6]	92.0 [75.1–113.3]	<0.01
Hemoglobin, g/dL [IQR]	14.2 [12.9–15.3]	12.9 [11.7–14.2]	14.6 [13.8–15.6]	<0.01
Thrombocytes, G/L [IQR]	232.0 [193.0–275.5]	228 [186–278]	233 [199–273]	0.31
Family History, n (%)	227 (21.0)	56 (15.8)	171 (23.6)	<0.01
Previous PCI, n (%)	319 (29.5)	109 (30.8)	210 (28.9)	0.57
Previous CABG, n (%)	115 (10.7)	46 (13.0)	69 (9.5)	0.09
Previous MI, n (%)	137 (12.7)	49 (13.8)	88 (12.1)	0.44
**Clinical presentation**				
Silent ischemia, n (%)	111 (10.3)	33 (9.3)	78 (10.7)	0.52
Stable angina, n (%)	222 (20.6)	76 (20.1)	146 (21.5)	0.63
Unstable angina, n (%)	114 (10.6)	29 (8.2)	85 (11.7)	0.09
NSTEMI, n (%)	259 (24.0)	71 (20.1)	188 (25.9)	0.04
STEMI, n (%)	245 (22.7)	67 (18.9)	178 (24.5)	0.04
Staged procedure, n (%)	20 (1.8)	10 (2.8)	10 (1.4)	0.15
Other, n (%)	109 (10.1)	68 (19.2)	41 (5.7)	<0.01
HAS-BLED score, mean ± SD	2.23 ± 0.97	2.92 ± 0.90	1.89 ± 0.79	<0.01
HEMMORR2HAGE score, mean ± SD	2.05 ± 1.37	3.12 ± 1.70	1.52 ± 0.76	<0.01
PARIS score, mean ± SD	4.44 ± 2.55	6.86 ± 2.45	3.26 ± 1.59	<0.01

*Continuous variables are expressed as mean ± SD, median [interquartile range] or number (%)*.

*BMI, body mass index; CABG, coronary artery bypass grafting; eGFR, estimated glomerular filtration rate; HBR, high bleeding risk; LBR, low bleeding risk; MI, myocardial infarction; PCI, percutaneous coronary intervention*.

The LBR group had a higher proportion of NSTEMI and STEMI. HAS-BLED, HEMMORR2HAGE and PARIS bleeding score were significantly higher in the HBR group.

In the whole patient population, at least 1 minor criterion was present in 456 patients (42.2%), while in the LBR group at least 1 minor criterion was found in 176 patients (24.4%). The most frequent minor HBR criterion was age >75 years (26.1%).

### Antithrombotic Regimens

The antithrombotic regimens were collected at hospital discharge and are summarized in Table 3. A DAPT therapy was initiated in 87.7%, of which aspirin + prasugrel was the most frequent combination (54.4%). As expected, there are significant differences between HBR and LBR groups with less DAPT (64.7 vs. 98.9%, *p* < 0.01), and half as much prasugrel use (*p* < 0.01) in the HBR (27.7%) compared to the LBR (67.4%) group. Triple antithrombotic therapy (TAT) was prescribed in 11.4% of patients. Interestingly, 1 (0.1%) LBR patient was given VKA after PCI. Since bleeding risk was assessed at time of PCI, even though the patient fulfilled a major HBR criteria at time of discharge, he was still considered in the LBR group.

**Table 3:** Antithrombotic regimen at hospital discharge.

	**All** ***N* = 1,080**	**HBR** ***N* = 354**	**LBR** ***N* = 726**	***p*-value**
DAPT, n (%)	947 (87.7)	229 (64.7)	718 (98.9)	<0.01
aspirin-clopidogrel, n (%)	264 (24.4)	116 (32.8)	148 (20.4)	<0.01
aspirin-prasugrel, n (%)	587 (54.4)	98 (27.7)	489 (67.4)	<0.01
aspirin-ticagrelor, n (%)	96 (8.9)	15 (4.2)	81 (11.2)	<0.01
TAT, n (%)	123 (11.4)	122 (34.5)	1 (0.1)	<0.01
DAPT-VKA, n (%)	69 (6.4)	68 (19.2)	1 (0.1)	<0.01
DAPT-DOAC, n (%)	54 (5.0)	54 (15.2)	0 (0)	<0.01
SAPT, n (%)	6 (0.6)	0 (0)	6 (0.8)	0.2
OAC + SAPT, n (%)	3 (0.3)	3 (0.8)	0 (0)	0.04
Only OAC, n (%)	1 (0.1)	0 (0)	1 (0.1)	1

*DAPT, dual antiplatelet therapy; DOAC, direct oral anticoagulant; HBR, high bleeding risk; LBR, low bleeding risk; TAT, triple antithrombotic therapy; VKA, vitamin K antagonist; SAPT, single antiplatelet therapy*.

### Clinical Endpoints

**Figure 1:**
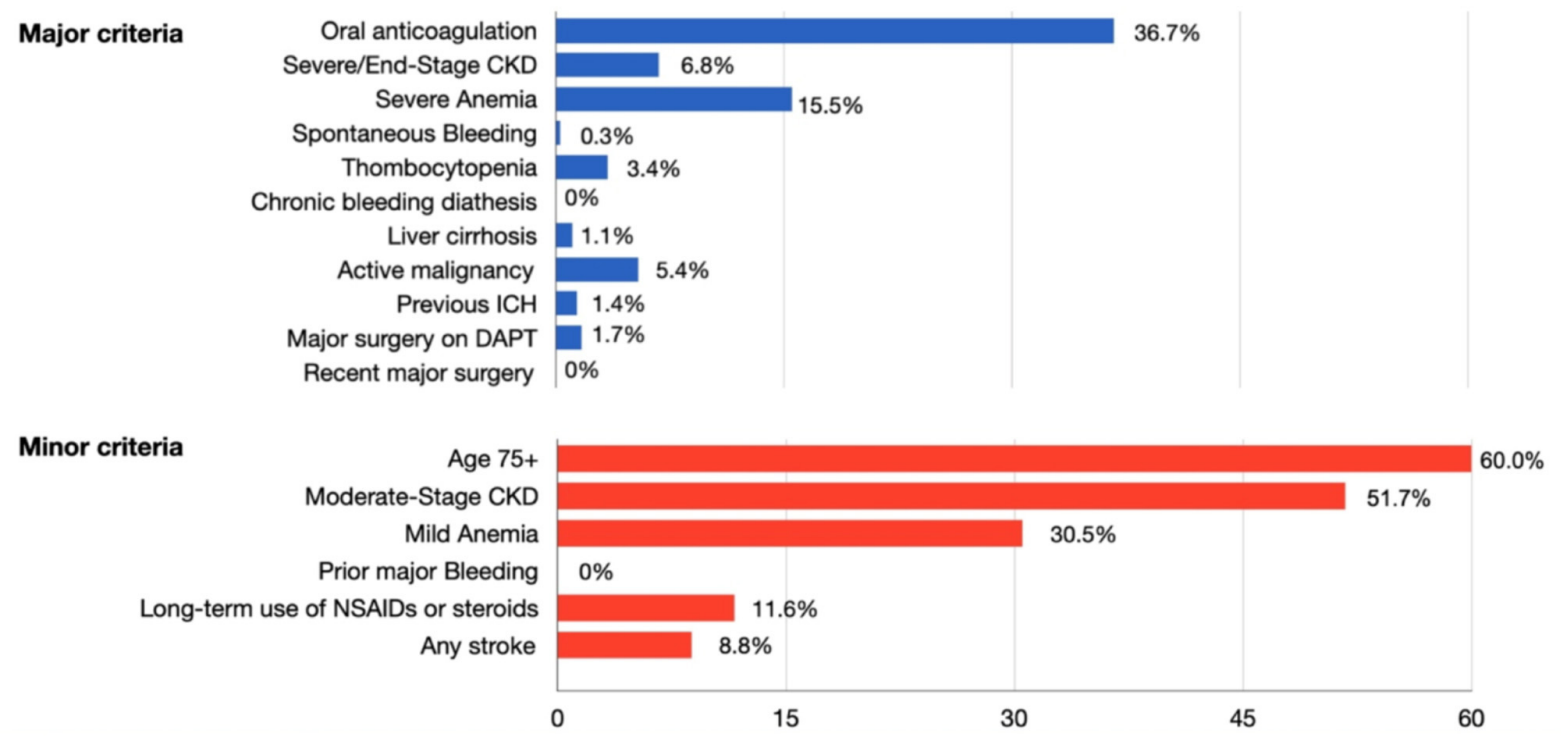
Prevalence of the ARC-HBR criteria in the HBR group. ARC, Academic Research Consortium; CKD, chronic kidney disease; DAPT, dual antiplatelet therapy; HBR, high bleeding risk; ICH, intracerebral hemorrhage; NSAIDs, non-steroidal anti-inflammatory drugs.

**Figure 2:**
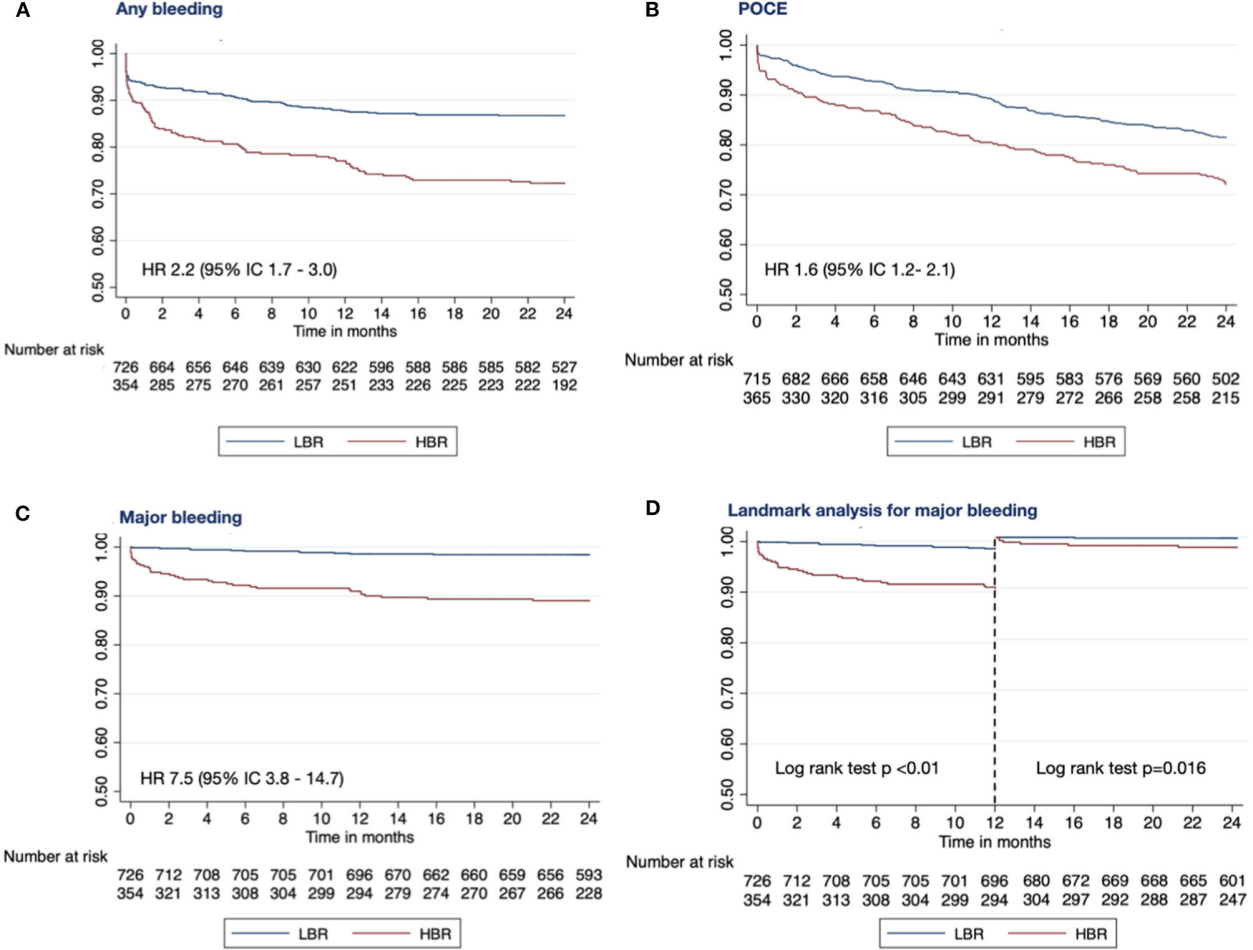
Kaplan-Meier survival curves showing any bleeding **(A)**, POCE [composite of all-cause death, any myocardial infarction and any repeat revascularization; **(B)**] and major bleeding **(C)** survival free at 2 years of follow-up. Landmark analysis **(D)** in patients with low-(LBR) and high-(HBR) bleeding risk. HBR, high bleeding risk; LBR, low bleeding risk; POCE, patient oriented composite endpoint.

Clinical endpoints are summarized in Table 4; Figures 1, 2.

**Table 4:** Clinical outcome at 2-year follow-up.

	**All** ***N* = 1,080**	**HBR** ***N* = 354**	**LBR** ***N* = 726**	***p*-value**
**Bleeding**				
Any bleeding, n (%)	189 (17.5)	94 (26.6)	95 (13.1)	<0.01
Cumulative incidence, in % (95% CI)	17.9 (1.2–15.7)	27.7 (23.3–32.8)	13.3 (1.3–11.0)	
Major bleeding (BARC 3-5) at 1 year, n (%)	41 (3.8)	31 (8.8)	10 (1.4)	<0.01
Cumulative incidence, in % (95% CI)	3.9 (0.3–5.2)	9.0 (6.4–12.6)	1.4 (0.8–2.6)	
Major bleeding (BARC 3–5) at 2 years, n (%)	48 (4.4)	37 (10.5)	11 (1.5)	<0.01
Cumulative incidence, in % (95% CI)	4.6 (3.5–6.0)	11.0 (8.1–14.8)	1.6 (0.9–2.8)	
**POCE**				
Any POCE, n (%)	230 (21.3)	100 (27.4)	130 (18.2)	<0.01
Any death, n (%)	72 (6.7)	51 (14.0)	21 (2.9)	<0.01
MI, n (%)	54 (5.0)	28 (7.7)	26 (3.6)	<0.01
Repeat revascularization, n (%)	150 (13.9)	49 (13.4)	101 (14.1)	0.99

*BARC, Bleeding Academic Research Consortium; CI, confidence interval; HBR, high bleeding risk; LBR, low bleeding risk; MI, myocardial infarction; POCE, patient-oriented composite endpoints*.

At 2-year follow-up, (17.5%) patients had a bleeding event, and (21.3%) an ischemic event. As anticipated, bleeding rates were higher in HBR compared to the LBR group with a 2.2-fold higher risk of any bleeding (26.6 vs. LBR 13.1%, *p* < 0.01) and a 7.5-fold higher risk of major bleeding (10.5 vs. LBR 1.5%, *p* < 0.01). Interestingly, one-third of major bleedings occurred during the first month after PCI.

### Multivariate Analysis of ARC-HBR Criteria

When included in the multivariable analysis, the major criteria mostly associated with BARC 3–5 major bleeding were moderate to severe anemia (HR 10.20 [5.39–19.30]; *p* < 0.01) and recent or recurrent major bleeding (HR 10.30 [1.30–82.19]; *p* = 0.03). With regards to minor criteria, major bleeding was mostly associated with age ≥75 year (HR 2.36 [1.19–4.70]; *p* = 0.01) and moderate renal failure (HR 2.36 [1.19–4.68]; *p* = 0.01). Overall, moderate to severe anemia was the ARC-HBR criterion associated with the highest risk of major bleeding complications at 2 years (Supplementary Table 1).

### Subgroup Analysis

**Figure 3:**
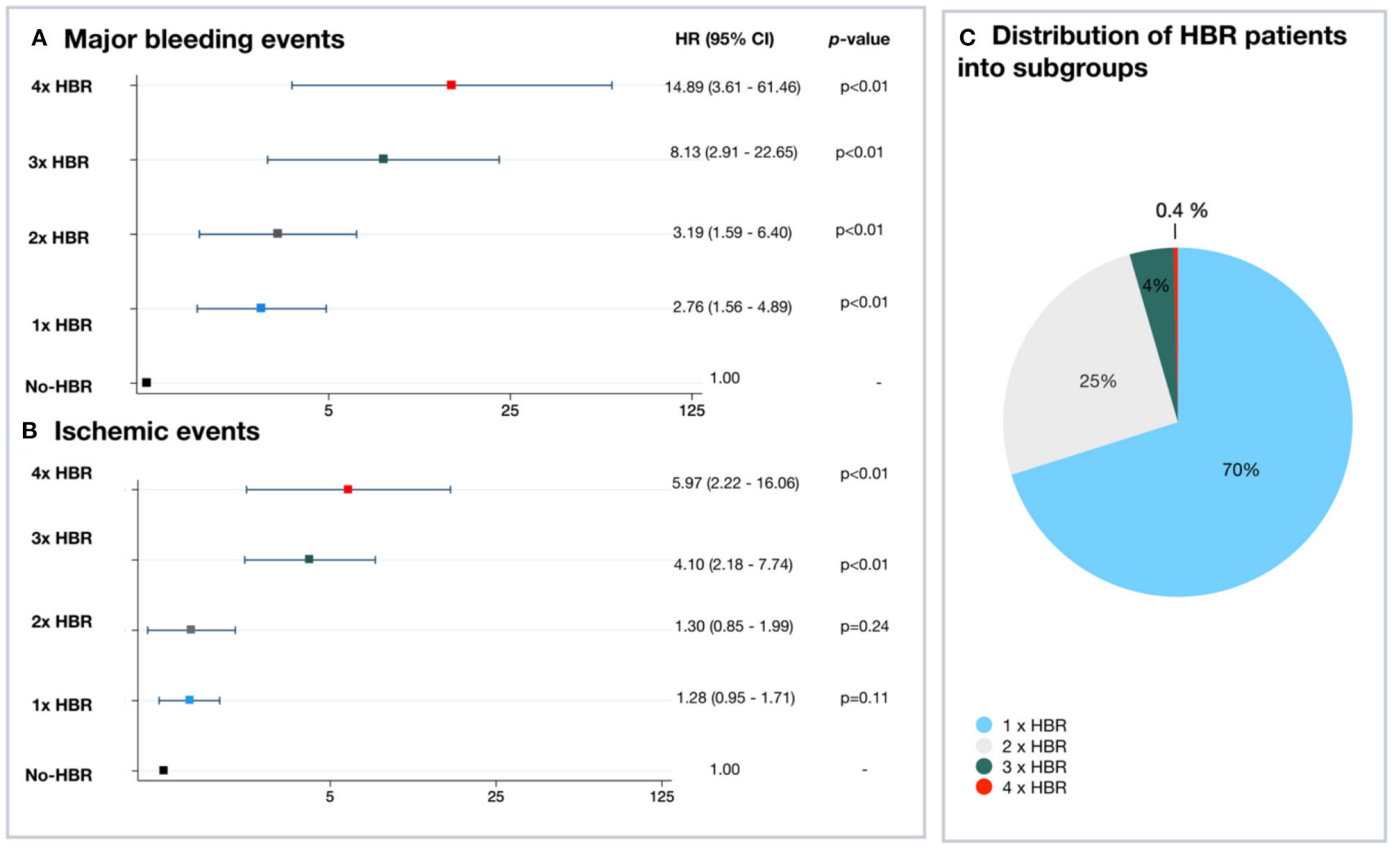
Incremental risk of major bleeding and ischemic endpoints according to the number of ARC-HBR criteria. **(A,B)** Risk of major bleeding and ischemic endpoints at 2 years for each HBR subgroup. **(C)** Distribution of HBR patients into subgroups. ARC, Academic Research Consortium; CI, confidence interval; HBR, high bleeding risk; HR, hazard ratio; LBR, low bleeding risk.

Among patients with multiple ARC-HBR criteria, major bleeding risk increases incrementally (one ARC-HBR criterion: 2.8-fold higher risk of bleeding; two ARC-HBR criteria: 3.2-fold higher risk of bleeding; three ARC-HBR criteria: 8.1-fold higher risk of bleeding, four ARC-HBR criteria: 14.9-fold higher risk of bleeding) (Figure 3).

## Discussion

The main results of this study are: (1) patients defined as HBR by the ARC-HBR definition are frequent in an unselected European population undergoing PCI; (2) the criteria defined by ARC-HBR accurately predict bleeding risk; (3) the impact of HBR risk factors is incremental; (4) one-third of major bleeding occurs during the first month after PCI; (5) the increased risk of bleeding is associated with an increased risk of ischemic events during a 2-year clinical follow-up.

Although a significant proportion of patients undergoing PCI are at high bleeding risk, they are often unrecognized and have also been excluded from many clinical trials (16). In a Japanese registry including 13,018 patients, Natsuaki et al. have observed that 43% fulfilled the ARC-HBR definition (17). The risk of bleeding at one year was 10.4% for HBR vs. 3.4% for the non-HBR group. They also demonstrated an incremental increase in GUSTO moderate to severe bleeding events according to the presence or absence of major and minor criteria. The risk of bleeding was 6.6% in patients without HBR-criteria, 14.7% with two minor HBR-criteria, 18.5% with one major HBR-criteria, 30.6% with two majors HBR-criteria, and 49.9% in patients with ≥3 major HBR-criteria. However, the use of GUSTO definition to define major bleedings, with a follow-up at one year, limits direct comparison with other registers.

Recently, Cao et al. (12) applied the ARC-HBR definition in a large cohort of 9,623 patients. The rate of primary bleeding endpoint at 1 year was 9.1% in HBR patients compared with 3.2% in non-HBR patients (*p* < 0.001). Not all the 20 ARC-HBR criteria were present in the analysis and reported severe bleeding events only partially overlapped with the BARC type 3 to 5 criteria.

We observed a similar rate of 32.8% of patients fulfilling the ARC-HBR definition. Our bleeding rates differ slightly with 8.8% of HBR patients presenting a major bleeding event vs. 1.4% in the LBR group. This may be in part explained by the inherent differences in baseline characteristics, DAPT strategies, bleeding definitions, and also the fact that not all major and minor criteria identified by the consortium were taken into consideration in the Japanese cohort.

In line with previous studies, we have observed a higher rate of POCE in HBR patients compared to LBR (18, 19). Overall, ACR-HBR patients may be at increased ischemic risk, as some risk factors present in the ARC-HBR definition are also global ischemic factors (20).

When considering DAPT duration strategies, it seems, that the bleeding risk may outweigh the ischemic risk in HBR patients, favoring shorter DAPT strategies (21). Eikelboom et al. have observed that 53% of bleeding in HBR and LBR patients occurred within the first month after PCI for acute coronary syndromes (22). Some patients will have a switch of DAPT therapy or anticoagulation which increases the risk of bleeding. Moreover, even short DAPT strategies recommend a minimum of 1-month DAPT. This vulnerable period may warrant a more specific and closer follow-up in HBR patients. We observed that one-third of patients bleed during the first month, which still implies that the majority of patients bleed after this critical period and underscores the importance of de-escalation of antiplatelet and antithrombotic therapies where appropriate.

Finally, we have observed an increased overall mortality in HBR patients compared to LBR patients. This observation may, in part, be driven by the fact that HBR patients have substantially more comorbidities than LBR patients. We did not observe any fatal bleeding events.

## Limitations

Our study is limited in size and conclusions should be interpreted as hypothesis-generating. This is was not a prospective assessment of the ARC-HBR definition, and the retrospective nature of the study raises the issue of unmeasured bias as well as incomplete data collection. However, patients with incomplete records were excluded from the study. One should be cautious in extrapolating the current results as this was a single-center study with homogenous practices amongst operators, a preponderant use of the femoral approach, and a majority of prasugrel as P2Y12 inhibitor for DAPT. We did not have data on DAPT adherence after discharge, and future research is important to understand how these factor influence bleeding rates in HBR patients. Another limitation is that some criteria are binary for simplification purposes, while the risk of bleeding and ischemic events is a continuum.

## Conclusion

The new ARC-HBR definition appropriately identified a population of patients at higher risk of bleeding after percutaneous coronary intervention. This increased risk of bleeding is associated with an increased risk of ischemic events during a 2-year clinical follow-up.

## Data Availability Statement

The raw data supporting the conclusions of this article will be made available by the authors, without undue reservation.

## Ethics Statement

The studies involving human participants were reviewed and approved by CER-VD (Lausanne). The patients/participants provided their written informed consent to participate in this study.

## Author Contributions

DD, ID, SS, DA, SeC, TH, PW, J-CS, J-JG, MT, SP, and StC have made substantial contributions to conception and design of the study, data acquisition, data analysis and interpretation, writing of the paper, and critical review for important intellectual content. All authors contributed to the article and approved the submitted version.

## Funding

The trial was an investigator-initiated study supported by an unrestricted grant from the Fonds Scientifique Cardiovasculaire (Fribourg, Switzerland).

## Conflict of Interest

The authors declare that the research was conducted in the absence of any commercial or financial relationships that could be construed as a potential conflict of interest.

## Publisher's Note

All claims expressed in this article are solely those of the authors and do not necessarily represent those of their affiliated organizations, or those of the publisher, the editors and the reviewers. Any product that may be evaluated in this article, or claim that may be made by its manufacturer, is not guaranteed or endorsed by the publisher.

## Abbreviations

BARC: Bleeding Academic Research Consortium
CKD: chronic kidney disease
DAPT: dual antiplatelet therapy
HBR: high bleeding risk
ARC-HBR: Academic Research Consortium High Bleeding Risk
LBR: low bleeding risk
MACE: major adverse cardiac events
MI: myocardial infarction
PCI: percutaneous coronary intervention
POCE: patient-oriented composite endpoints
TAT: triple antithrombotic therapy.
