# Supplementary material for: Ischemic and Bleeding Outcomes According to the Academic Research Consortium High Bleeding Risk Criteria in All Comers Treated by Percutaneous Coronary Interventions
Source: Front Cardiovasc Med. 2021 Dec 2;8:620354. doi: 10.3389/fcvm.2021.620354 (PMC8674503; doi:10.3389/fcvm.2021.620354)
Supplement: Supplementary file 1 [file Data_Sheet_1.docx]

**Supplementary Figure 1.** Study flow-chart
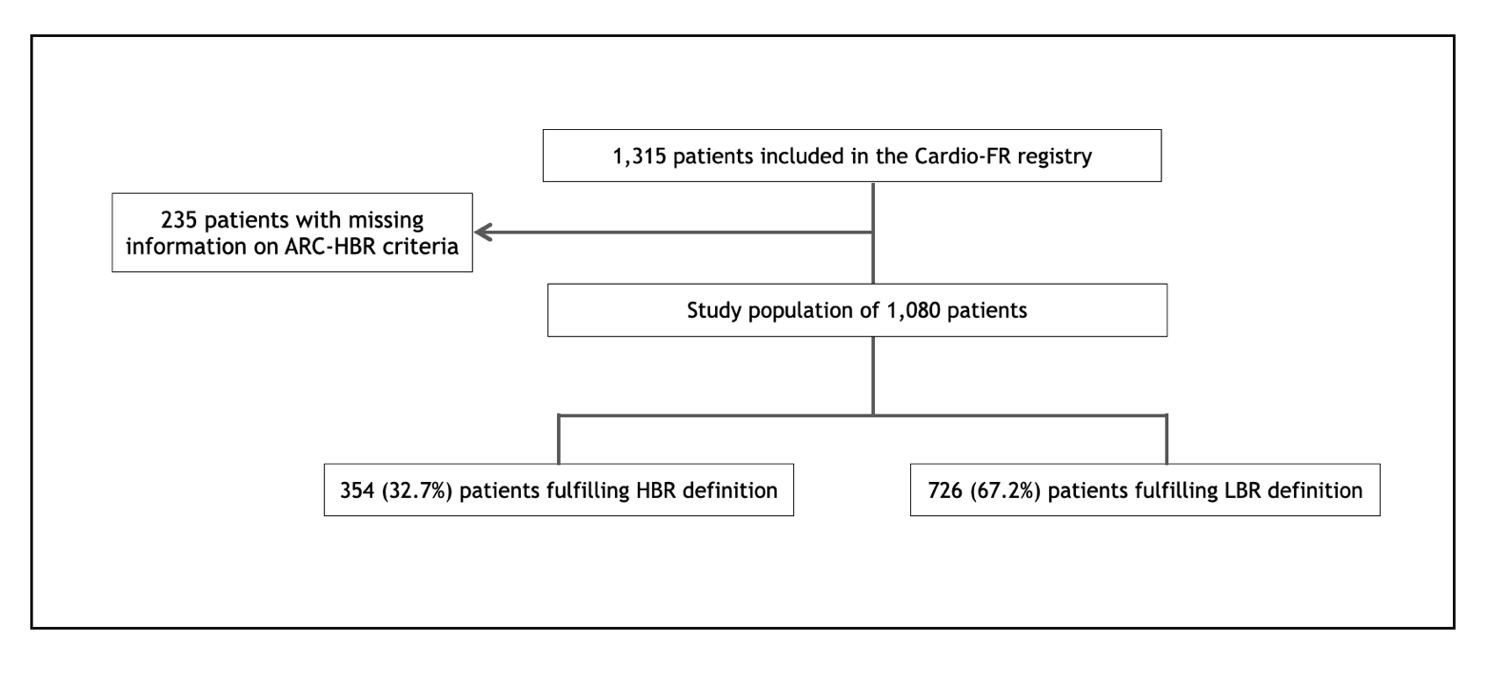


ARC-HBR, Academic Research Consortium for High Bleeding Risk; HBR, high bleeding risk; LBR, low bleeding risk.
