# Supplementary material for: Ischemic and Bleeding Outcomes According to the Academic Research Consortium High Bleeding Risk Criteria in All Comers Treated by Percutaneous Coronary Interventions
Source: Front Cardiovasc Med. 2021 Dec 2;8:620354. doi: 10.3389/fcvm.2021.620354 (PMC8674503; doi:10.3389/fcvm.2021.620354)
Supplement: Supplementary file 2 [file Table_1.docx]

**Supplementary Table 1.** Risk of the major bleeding endpoint at 2 year according to each major or minor ARC-HBR criteria

| ARC-HBR criteria | Multivariate HR  (95% CI) | *p*-value |
| --- | --- | --- |
| Major criteria |  |  |
| Oral anticoagulation | 1.88 (0.98-3.60) | 0.06 |
| Moderate/severe anemia | 10.20 (5.39-19.30) | <0.01 |
| Spontaneous bleeding | 10.30 (1.30-82.19) | 0.03 |
| Previous ICH | 5.60 (0.75-41.72) | 0.09 |
| Minor criteria |  |  |
| Age 75+ | 2.36 (1.19-4.69) | 0.01 |
| Moderate CKD | 2.36 (1.19-4.68) | 0.01 |
| Mild anemia | 0.96 (0.46-2.02) | 0.92 |
| Prior CVA | 0.40 (0.05-2.89) | 0.36 |

ARC-HBR, Academic Research Consortium for High Bleeding Risk; CKD, chronic kidney disease; CVA, cerebrovascular accident; 75+, ≥75 years; DAPT, dual antiplatelet therapy; ICH, intracerebral hemorrhage ; NSAID, nonsteroidal anti-inflammatory drugs.
